# WASp Deficiency Selectively Affects the TCR Diversity of Different Memory T Cell Subsets in WAS Chimeric Mice

**DOI:** 10.3389/fimmu.2021.794795

**Published:** 2022-01-18

**Authors:** Wenyan Li, Yanjun Jia, Yanping Wang, Qin Zhao, Lu Yang, Ting Zeng, Linlin Niu, Rongxin Dai, Yanan Li, Xiaodong Zhao, Junfeng Wu

**Affiliations:** ^1^ National Clinical Research Center for Child Health and Disorders, Ministry of Education Key Laboratory of Child Development and Disorders, Chongqing Key Laboratory of Child Infection and Immunity, Chongqing Key Laboratory of Pediatrics, Children’s Hospital of Chongqing Medical University, Chongqing, China; ^2^ Department of Rheumatology and Immunology, Children’s Hospital of Chongqing Medical University, Chongqing, China

**Keywords:** Wiskott–Aldrich Syndrome, memory T cell, T cell receptor repertoire, high-throughput sequencing, chimeric mouse model

## Abstract

**Background:**

The T cell receptor (TCR) diversity is essential for effective T cell immunity. Previous studies showed that TCR diversity in Wiskott–Aldrich Syndrome (WAS) patients was severely impaired, especially in the memory T cell populations. Whether this defect was caused by intrinsic WASp deficiency or extrinsic reasons is still unclear.

**Methods:**

We sorted different T cell subsets from the bone marrow chimeric mice model using both magnetic beads and flow cytometry. TCR repertoires of memory T cells, especially CD4^+^ effector memory T (TEM) cells and CD8^+^ central memory T (TCM) cells, were analyzed using the UMI quantitative high-throughput sequencing (HTS).

**Results:**

An average of 5.51 million sequencing reads of 32 samples was obtained from the Illumina sequencing platform. Bioinformatic analyses showed that compared with wild type (WT), WAS knock out (KO)-CD4^+^ TEM cells exhibited increased Simpson index and decreased D50 index (P <0.05); The rank abundance curve of KO-CD4^+^ TEM cells was shorter and steeper than that of WT, and the angle of ^q^D and q in KO-CD4^+^ TEM cells was lower than that of WT, while these indexes showed few changes between WT and KO chimeric mice in the CD8^+^TCM population. Therefore, it indicated that the restriction on the TCRVβ repertoires is majorly in KO-CD4^+^ TEM cells but not KO- CD8^+^ TCM cells. Principal Component Analysis (PCA), a comprehensive parameter for TCRVβ diversity, successfully segregated CD4^+^ TEM cells from WT and KO, but failed in CD8^+^ TCM cells. Among the total sequences of *TRB*, the usage of TRBV12.2, TRBV30, TRBV31, TRBV4, TRBD1, TRBD2, TRBJ1.1, and TRBJ1.4 showed a significant difference between WT-CD4^+^ TEM cells and KO-CD4^+^ TEM cells (P <0.05), while in CD8^+^ TCM cells, only the usage of TRBV12.2 and TRBV20 showed a substantial difference between WT and KO (P <0.05). No significant differences in the hydrophobicity and sequence length of TCRVβ were found between the WT and KO groups.

**Conclusion:**

WASp deficiency selectively affected the TCR diversity of different memory T cell subsets, and it had more impact on the TCRVβ diversity of CD4^+^ TEM cells than CD8^+^ TCM cells. Moreover, the limitation of TCRVβ diversity of CD4^+^ TEM cells and CD8^+^ TCM cells in WAS was not severe but intrinsic.

## Introduction

Wiskott–Aldrich syndrome protein (WASp) is expressed exclusively in the hematopoietic cells, consisting of five main functional domains, a WASp-homology 1/pleckstrin homology (WH1/PH) domain, a basic domain, a GTPase binding domain (GBD), a proline-rich region, and a C-terminal VCA region (a verproline (V) homology domain, a cofilin (C) homology domain; and a central acidic (A) region) which binds the Arp2/3 complex enhancing actin nucleation and rapid formation of new actin filaments (1, 2). As an actin nucleation promoting factor, WASp regulates the structure and dynamics of actin filament networks of the cells (3). The absence or altered structures of WASp result in the Wiskott–Aldrich syndrome (WAS), a rare primary immunodeficiency disease, which is clinically characterized by thrombocytopenia, eczema, immunodeficiency, and increased risk of autoimmune diseases and lymphoid malignancies (4). Indeed, numerous cellular activities of the immune system have been described to be affected in WAS patients, such as reduced chemotactic responses and phagocytic abilities of monocytes and macrophages, impaired activation, differentiation, and proliferation of multiple T and B lymphocyte subsets (4–7). Abnormal T cell functions caused by WASp-deficiency mainly lead to immune deficiency in patients with WAS. The abnormal T cell functions in WAS patients include T lymphopenia, which gradually aggravated with age, decreased immune synapse formation, reduced synthesis, secretion of T cell cytokines (such as IL-2, IFN-γ, and TNF-α), impaired function of cytotoxic T cells, abnormal chemotaxis of T cells *in vitro*, and dysfunction of Treg and regulatory helper T cells (5, 8–11).

T cell receptor (TCR) diversity is an essential guarantee for effective T cell immunity. The TCR repertoire is composed of all TCR clones, in which each TCR clone specifically recognizes the corresponding antigen. The abundance of TCR diversity determines the potential of T cell response to various antigens in the changeable environment. Recombination of Variable (V), Diversity (D), and Joining (J) gene elements allow the establishment of TCR repertoire (12, 13). With the fast-developing next-generation sequencing technology, several studies have explored the role of WASp in the TCR recombination process. In 2005, Wada et al. firstly studied the diversity of TCR in WAS patients and found that TCRVβ repertoire was specifically skewed in WAS patients older than 15 years old (14). Then, Braun et al. and our team confirmed that young WAS patients also had a TCRVβ repertoire defect (15, 16). We further found that the TCR diversity of WAS patients was severely limited in memory/effector CD4^+^ T cells and terminal effector CD8^+^ T cells. In contrast, naïve CD4^+^ T cells and naïve CD8^+^ T cells showed no limitation on TCR diversity. O’Connell et al. also showed WAS patients had TCR clonal expansion in memory CD4^+^ T cells, naïve and memory CD8^+^ T cells (17).

Previous studies have confirmed that the number of TCR clones is affected by many factors, such as age, pathogen infection, tumor, autoimmune diseases, immunization, and immunosuppression (18, 19). Petersen et al. showed that TCR diversity was limited in old WASp^−/−^ mice, but not in young WASp^−/−^ mice. They suggested that autoantigens are likely the cause of reduced TCR diversity in WAS in the absence of infections (20). However, whether the TCR diversity limitation in WAS was caused by intrinsic WASp deficiency is still unclear. Here, we further explored the impact of WASp on TCR diversity of different memory T cell subsets in WAS chimeric mice by unique molecular identifiers (UMI) quantitative high-throughput sequencing (HTS) technology. Our data indicated that WASp deficiency had more impact on the TCRVβ variety of CD4^+^ TEM cells than that of CD8^+^ TCM cells. Moreover, the limitation on the TCRVβ diversity of CD4^+^ TEM cells and CD8^+^ TCM cells in WAS is not severe but intrinsic. It provides valuable information for unraveling the role of WASp in the TCR recombination process.

## Materials and Methods

### Mouse strains and Chimeric Mice by Bone Marrow Transplantation

Wenxia Song from the University of Maryland kindly provided WASp-KO mice expressing CD45.2 on the C57BL/6 background. WT C57BL/6 mice expressing CD45.1 were purchased from Shanghai Model Organisms. For a generation of bone marrow (BM) chimeras, a total of 5 × 10^6^ BM cells containing WT CD45.1 and WT or WASp*^−/−^* CD45.2 at a 1:3 ratio were injected into lethally irradiated (6 Gy) WT CD45.1 recipient animals *via* tail vein. All donor mice were 6–8 weeks old. Chimeric mice were analyzed 10 weeks after transplantation (21). All animal work was reviewed and proved by the Institutional Animal Care and Usage Committee of Children’s Hospital of Chongqing Medical University.

### Cell Sorting

Purified T cells from chimeric mice were isolated by immunomagnetic negative selection (Stem Cell Technologies, Canada, Cat. 19751). Then, they were stained with the following antibodies: anti-CD3-FITC, anti-CD4-PE/CY7, anti-CD8-APC, anti-CD44-perCP/CY5.5, anti-CD62L-BV421, anti-CD45.1-BV510, and anti-CD45.2-APC-CY7. CD4^+^ effector memory T cells (CD45.2^+^CD4^+^CD44^hi^CD62L^low^) and CD8^+^ central memory T cells (CD45.2^+^CD8^+^ CD44^hi^CD62L^hi^) were then sorted using a FACSAria II (BD Biosciences) (**Figure S1**). All antibodies were purchased from BioLegend, USA. The purity of sorted cell subtypes exceeded 95%, as assessed by flow cytometry analysis. **Table S1** lists the proportions and actual collected cell numbers of the two sorted subsets in each sample.

### High-Throughput TCR Repertoire Sequencing and Bioinformatic Analyses

RNA was isolated using the RNeasy Mini Kit (Qiagen, Germany) following the manufacturers’ instructions and was sent to Huayin Health Technology Co., Ltd. (Guangzhou, China) for HTS analysis employing unique molecular identifiers (UMI) (Publication Patent Number: CN108893464A). RNA concentrations were determined using a NanoDrop 2000 spectrophotometer (Thermo Scientific). cDNA libraries contained UMI for HTS were prepared by 5’ rapid amplification of cDNA ends (RACE) using the single primer designed according to the constant region. Then, two rounds of nested PCR were performed for TCRVβ library preparation and the products were purified using QIAquick PCR Purification Kit (Qiagen, Germany). According to the manufacturer’s protocol, Illumina adaptors were ligated using the NEBnext Ultra DNA Library Prep kit (New England BioLabs, USA). Then products were identified on 2% agarose gels, and bands centered at 600–800 bp were excised and purified using a QIAquick Gel Extraction kit (Qiagen, Germany). The purified PCR product was subjected to HTS using the Illumina HiSeqX Ten (PE150) and HiSeqX Ten Reagent kit v2.5 (FC-501-2501). Low-quality sequences were discarded. TCRβ V, D, and J gene identification, CDR3 sequence extraction and error corrections in clean reads were performed using miTCR.

Considering the influence of differences in sample size on diversity indices, we randomly sampled 4,000, 6,000, 8,000, 10,000, and 12,000 UMI from each sample for this analysis. Shannon, Simpson (1-D), D50, Chao 1, TOP100, and ^q^D were assessed based on previously published work (22, 23). Overlap indices were calculated by the overlap coefficient (overlap (X, Y) = |X and Y|/min (|X|, |Y|) for nucleotide sequences (species = nucleotide sequence) (24). TCR CDR3 overlap was assessed by ‘F2’, ‘R’ and ‘D’ metrics in VDJTOOLS software (25). The similarity of CDR3 amino acid was assessed by Bhattacharyya distance as previously described (26). The CDR3 nucleotide length was assessed by Complexity score and Skewness (22). The hydrophobic index was calculated by the frequency of hydrophobic amino acid doublets at positions 6 and 7 of the CDR3β (22, 27). Cysteine index was calculated by the frequency of TCRVβ sequences with cysteine within 2 positions of the CDR3 (27).

### Statistical Analysis

The Student’s t-test was used to compare diversity parameters in different groups. The Chi-squared test was used to compare groups in analysis involving qualitative variables. The Wilcoxon rank-sum test was used to compare independent samples. Dunnett’s multiple comparisons were used for multiple t-tests. Data analysis was performed by GraphPad Prism 7.0 (GraphPad Software, San Diego, CA); p-value <0.05 was considered statistically significant.

## Results

To investigate whether WASp creates diverse TCR repertoires in memory T cells independent of the influence from infection and homeostasis, we established BM chimeras of WT (CD45.1) and KO (WASp*^−/−^* CD45.2). For HTS, WT or KO CD4^+^ effector memory T (CD4^+^ TEM) cells and CD8^+^ central memory T (CD8^+^ TCM) cells were sorted 10 weeks after transplantation (21). The proportion and the actual number of collected CD4^+^ TEM and CD8^+^ TCM are shown in **Table S1**. Due to a limited cell number, CD4^+^ central memory T (CD4^+^ TCM) cells and CD8^+^ effector memory T (CD8^+^ TEM) cells were not included in the HTS analysis.

Since comparative analysis requires accurate normalization, unique molecular identifiers (UMI) were used to process sequencing data. In total, we obtained an average of 5.51 million sequencing reads from 32 samples using the Illumina sequencing platform. On average, 79.15% (range from 69.38 to 88.7%) of these sequence reads were utilized after filtering out low-quality ones. The number of total and unique sequences, total and unique clones and clone types of rearranged TCRVβ products for each sample is listed in **Table S2**. In particular, the number of unique CDR3 sequences, unique CDR3aa and clone types in KO-CD4^+^ TEM cells showed a downward trend, but no statistical difference was observed compared to WT.

### Light Restriction on the TCRVβ Repertoires in WASp*^−^* ^/^ *^−^* CD4^+^ TEM Cells but not in WASp*^−^* ^/^ *^−^* CD8^+^ TCM Cells

**Figure 1:**
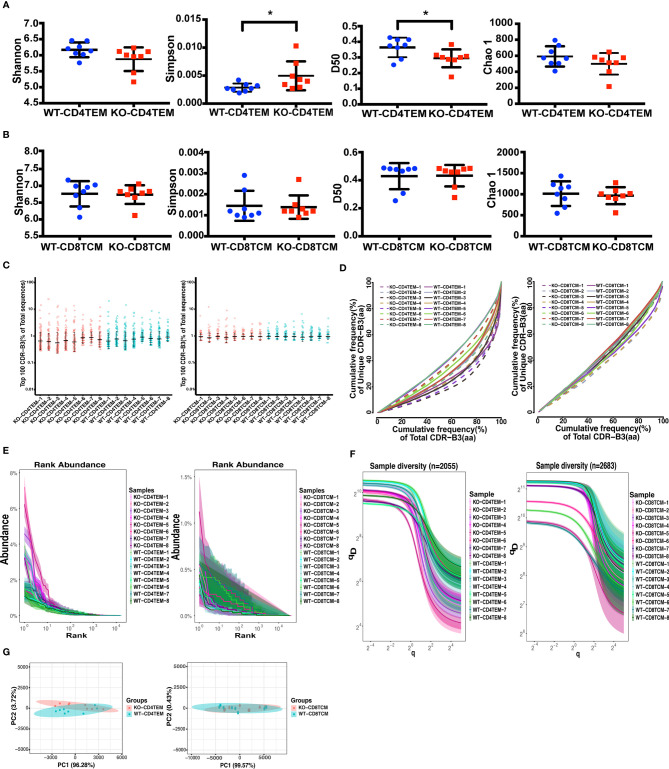
Diversity and clonality analysis of TCRVβ repertoires of CD4^+^ TEM cells and CD8^+^ TCM cells in WT and KO chimeras. Quantification of the diversity, unevenness, clonotypic expansion, and richness of TCRVβ repertoires using the Shannon–Wiener index, Simpson index, D50, and Chao 1 index in CD4^+^ TEM cells **(A)** or CD8^+^ TCM cells **(B)**. Representation of the frequency of the top 100 most abundant clones for *TRB* sequences **(C)**. The cumulative frequencies of unique versus total CDR3 clonotypes are shown for TCRβ repertoires **(D)**. Mean values ± SE are shown; t-test was used for statistical analysis, *p <0.05. Showing is the Rank-abundance curve and sample diversity curve by using the abundance of total *TRB* sequences versus *TRB* sequences Rank **(E)** and true diversity (^q^D) versus q **(F)**. Sample plots illustrating the segregation of the various KO from WT chimeras based on primary component (PC) 1 and 2 determined by four variables (Shannon–Wiener index, Simpson, number of total and unique sequences) for TCRVβ repertoires **(G)**.

TCRVβ repertoire diversity and clonality were assessed using several widely used diversity parameters: Shannon–Wiener index, Simpson index, D50, and Chao 1 index. Consistent with our findings in WAS patients, TCR repertoire was selectively skewed in CD45RO^+^CD4^+^ T cells. Compared to WT, KO-CD4^+^ TEM cells had a higher Simpson index and lower D50 diversity index, indicating an unequal distribution of clonotypes and more clonotypic expansions in KO-CD4^+^TEM cells. There was no statistical difference in the Shannon index and Chao 1 index, representing the comparable richness and abundance between WT and KO in CD4^+^ TEM cells (**Figure 1A**). No difference between WT and KO in CD8^+^ TCM cells for these four parameters was found (**Figure 1B**). In addition, clonal expansion was further assessed by the cumulative frequencies of unique versus total CDR3 clonotypes and the Top 100, which corresponds to the percentage of top 100 CDR3 sequences in the total number of sequences. At the same time, the results showed no marked difference between WT and KO in CD4^+^ TEM cells or CD8^+^ TCM cells (**Figures 1C, D** **)**. To further analyze the abundance and sample diversity, we estimated Rank abundance and true diversity (^q^D) by observing their corresponding curves. The abundance curve of KO-CD4^+^ TEM cells was steeper and shorter, and ^q^D curves of KO-CD4^+^ TEM cells were clearly lower than that of WT, therefore suggesting WASp deficiency decreased the uniformity and diversity of TCRVβ repertoire in CD4^+^ TEM cells. In comparison, the rank abundance and sample diversity curves of CD8^+^ TCM cells in WT and KO were almost overlapped (**Figures 1E, F** **)**.

To assess whether analysis of the TCRVβ repertoire of CD4^+^ TEM cells and CD8^+^ TCM cells may distinguish KO chimeras from WT, we used Principal Component Analysis (PCA) based on four variables: Shannon–Wiener index, Simpson, the number of total and unique sequences. As expected, PCA successfully segregated CD4^+^ TEM cells from WT and KO but failed to discriminate between CD8^+^ TCM cells from WT and KO (**Figure 1G**). Collectively, WASp-deficiency slightly affects the TCRVβ diversity of CD4^+^ TEM cells in the chimeric mice model, but not the CD8^+^ TCM cells.

### Skewed Usage of V, D and J Segment Genes in WASp*^−^* ^/^ *^−^* Memory T Cells

**Figure 2:**
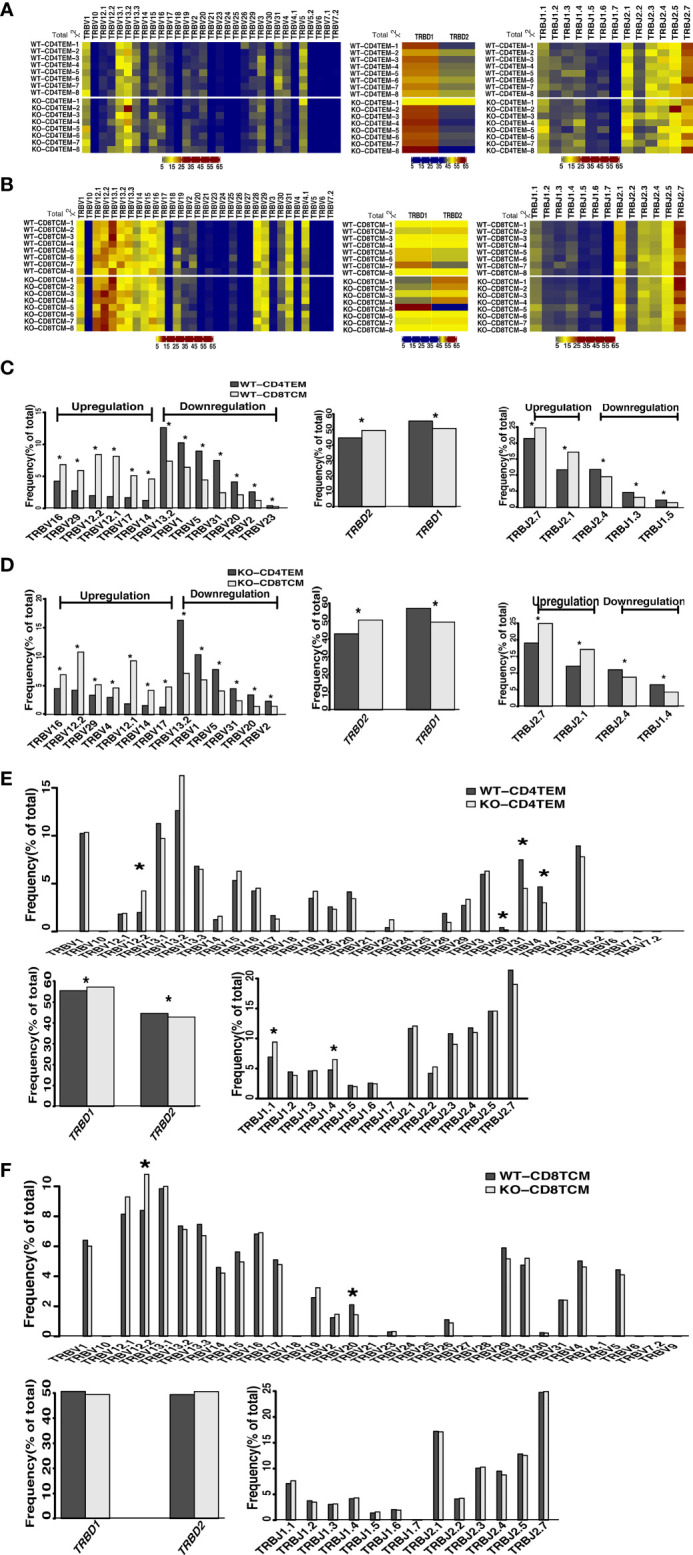
Differential usage of V, D and J genes in the total sequences of *TRB* repertoires of CD4^+^ TEM cells and CD8^+^ TCM cells in WT and KO chimeras. Heatmap represents V, D and J gene usage frequency for total *TRB* sequences of CD4^+^ TEM cells **(A)** and CD8^+^ TCM cells **(B)** in WT and KO chimeras. Relative frequency for the usage of TRBV, TRBD, and TRBJ gene segments for CD4^+^ TEM cells vs CD8^+^ TCM cells in WT chimeras **(C)**, CD4^+^ TEM cells vs CD8^+^ TCM cells in KO chimeras **(D)**, WT vs KO chimeras in CD4^+^ TEM cells **(E)** and WT vs KO chimeras in CD8^+^ TCM cells **(F)**. *p < 0.05.

The *V*(*D)J* recombination is the first determinant of TCR diversity. Analysis of *TRB* sequences composition helps understand the usage of individual *V*, *D*, and *J* elements. As shown in the heat map, we analyzed the proportion of *V*, *D* and *J* segment genes among the total sequences of *TRB*. We found no apparent non-stochastic restriction on the usage of *V*, *D* and *J* segments in KO chimeras compared with WT (**Figures 2A, B** **)**. Then, we further compared each *V*, *D* and *J* subfamily genes and found significant differences in the usage of V, D and J segments between CD4^+^ TEM cells and CD8^+^ TCM cells in both WT and KO chimeras as expected (**Figures 2C, D** **)**. However, compared to CD4^+^ TEM cells, the upregulation of TRBV4 and the downregulation of TRBJ1.4 usage in CD8^+^ TCM cells were found explicitly in KO chimeras. In contrast, the downregulation of TRBV23, TRBJ1.3, and TRBJ1.5 were specifically found in WT chimeras (**Figures 2C, D** **)**. The usage of TRBV12.2 and TRBD1 was upregulated, and that of TRBV30, TRBV31, TRBV4, TRBD2, TRBJ1.1, and TRBJ1.4 was downregulated when comparing CD4^+^ TEM cells in WT and KO (**Figure 2E**). As for the comparison of CD8^+^ TCM cells in WT and KO, the usage of TRBV12.2 was increased, TRBV20 was decreased, and D and J segments showed no difference (**Figure 2F**). We also analyzed the composition of the unique sequences of *TRB*, and the results were almost consistent with those in the total sequence (**Figure S2**). Thus, WASp deficiency disturbed the usage of V, D and J genes of CD4^+^ TEM cells, and V gene segments of CD8^+^ TCM cells.

### Altered Combinations of V(D)J Genes in WASp*^−^* ^/^ *^−^* CD4^+^ TEM Cells and WASp*^−^* ^/^ *^−^* CD8^+^ TCM Cells

**Figure 3:**
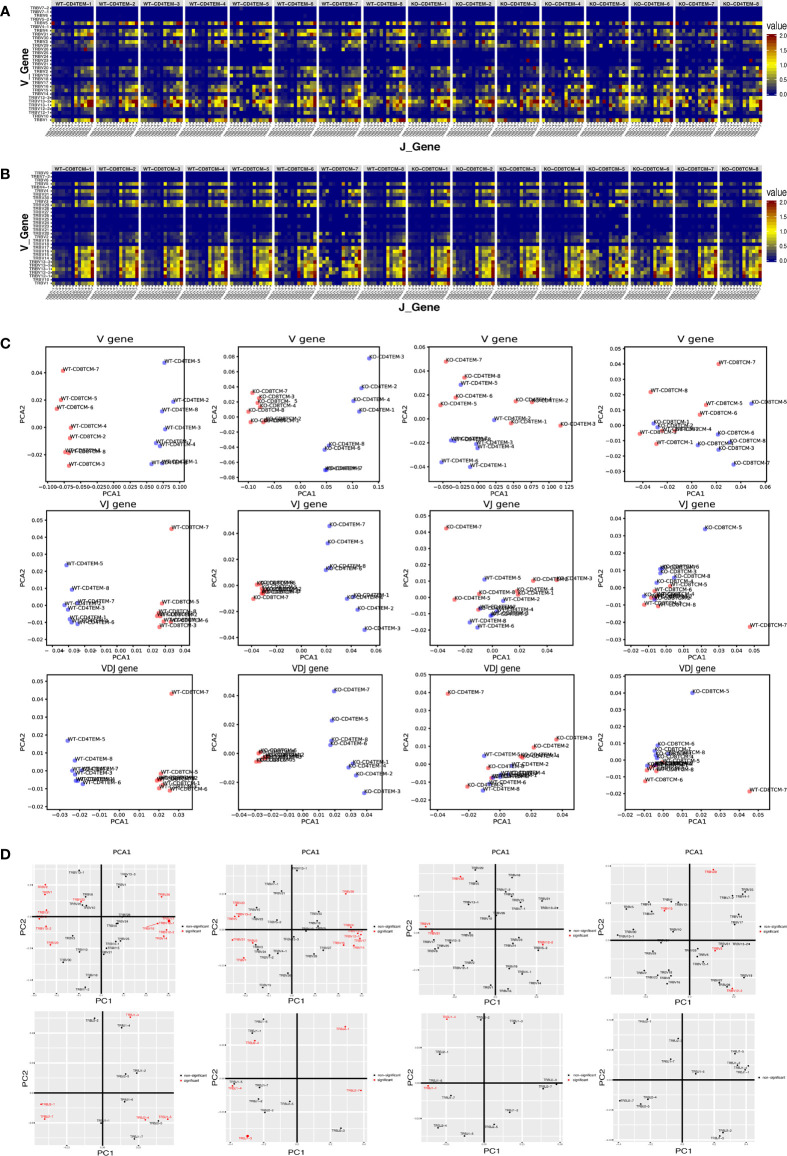
Differential V–J and V–D–J combination of CD4^+^ TEM cells and CD8^+^ TCM cells in WT and KO chimeras. Heatmap representing the frequency of V–J combination for CD4^+^ TEM cells **(A)** and CD8^+^ TCM cells **(B)** in WT and KO chimeras. Sample plots illustrating the segregation of CD4^+^ TEM cells from CD8^+^ TCM cells in WT chimeras, CD4^+^ TEM cells from CD8^+^ TCM cells in KO chimeras, KO from WT chimeras in CD4^+^ TEM cells, and that of KO from WT chimeras in CD8^+^ TCM cells (from left to right) based on PCA of V genes, the combination of V–J and also V–D–J, distribution of V gene families and J gene families (from up to down) **(C)**. V and J gene families with significant differences were shown in red **(D)** (p < 0.05).

To further explore whether WASp participates in the combination of V(D)J genes, we analyzed the combination of individual V, D and J genes in total *TRB* sequences. In the combination of V and J genes, the lower right part of KO-CD4^+^ TEM group was more cluttered compared to the WT group, suggesting that some combination of V–J genes in CD4^+^ TEM cells was altered in KO chimeras (**Figure 3A**). In contrast, the difference between WT and KO in CD8^+^ TCM cells was not apparent (**Figure 3B**). To assess overall differences among each individual, we used PCA analysis based on V gene segments, V–J genes combination or V(D)J genes combination to display four compare groups. The pictures showed that V genes and the combination of V–J genes as well as V(D)J genes of CD4^+^ TEM cells and CD8^+^ TCM cells could be clearly distinguished into two groups in WT and KO chimeras. In contrast, the group of WT-CD4^+^ TEM cells was more consistent than KO-CD4^+^ TEM. WT and KO chimeras are distinguishable from the PCA analysis based on V genes in both CD4^+^ TEM cells and CD8^+^ TCM cells. And the PCA analysis based on the combination of V–J genes and V(D)J genes still can be divided into two groups of WT and KO chimeras in both CD4^+^ TEM cells and CD8^+^ TCM cells, but less difference was found than that based on V genes (**Figure 3C**). We also showed the analysis of PCA based on the V gene segments and J gene segments. The difference in CD4^+^TEM cells between WT and KO is attributed more to the selection of TRBV12.2, TRBV30, TRBV31, TRBV4, TRBJ1-1, and TRBJ1-4 genes, while the distinguishable clustering of CD8^+^TCM cells between KO and WT was more influenced by the selection of TRBV12.2, TRBV15, TRBV20, and TRBV3 genes (**Figure 3D**).

### The Higher Similarity of TCRVβ Repertoires of WASp*^−^* ^/^ *^−^* CD4^+^TEM Cells and CD8^+^ TCM Cells

**Figure 4:**
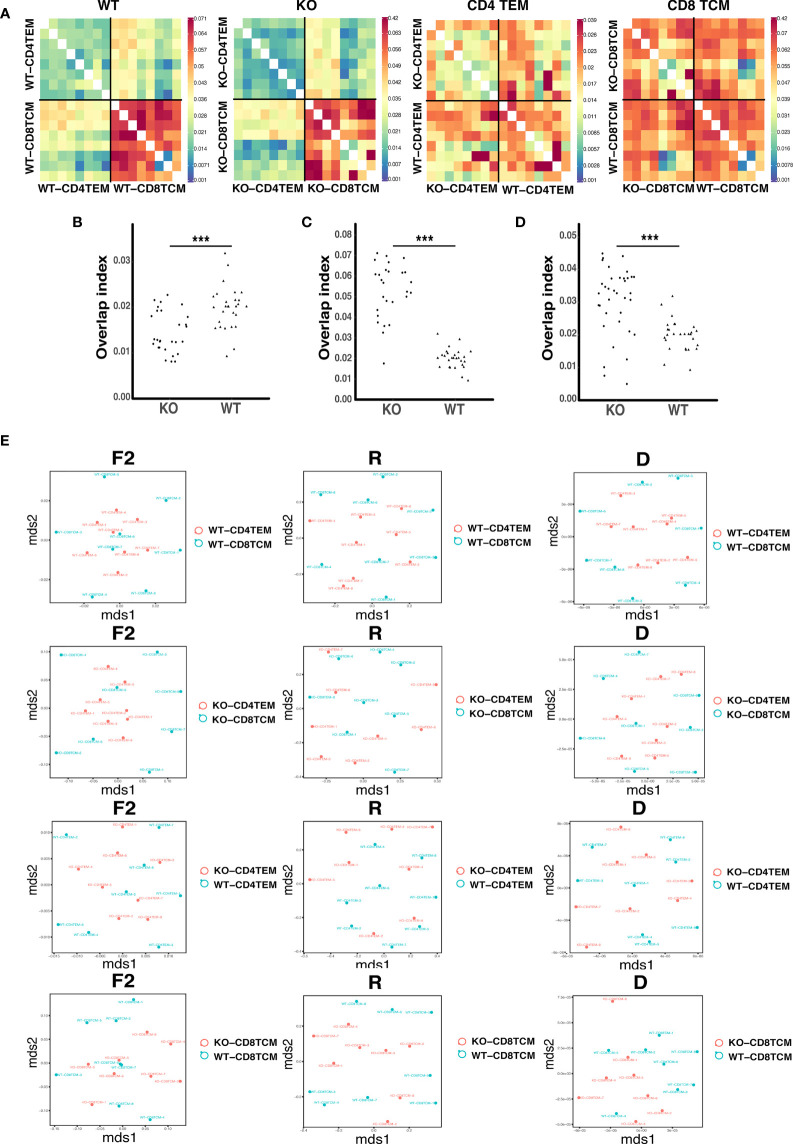
The relative similarity of TCRVβ repertoires in WT and KO chimeric mice. Heatmap represents the distance matrix of TCRVβ repertoires of CD4^+^ TEM and CD8^+^ TCM cells in WT and KO chimeric mice **(A)**. Overlap indices for CD4^+^ TEM/CD4^+^ TEM **(B)**, CD8^+^ TCM/CD8^+^ TCM **(C)** and CD4^+^ TEM/CD8^+^ TCM **(D)**. Metrics F2, R and D for CD4^+^ TEM and CD8 TCM TRB sequences in WT and KO chimeric mice **(E)**. ***p < 0.001.

The highly shared TCR repertoires are enriched in clonotypes bearing fewer insertions and were reported in autoimmune diseases like type 1 diabetes (24). To detect the degree of sequence sharing, we calculated overlap indices for TCRVβ repertoires of CD4^+^ TEM cells and CD8^+^ TCM cells. As presented in the distance heat map, CD4^+^ TEM cells and CD8^+^ TCM cells were more similar among KO chimeras than WT. WT and KO were more similar among CD4^+^ TEM cells than CD8^+^ TCM cells (**Figure 4A**). The value of overlap indices for CD4^+^ TEM/CD4^+^ TEM was lower (**Figure 4B**), while the one for CD8^+^ TCM/CD8^+^ TCM was higher in KO than WT (**Figure 4C**). The one for CD4^+^ TEM/CD8^+^ TCM in KO was increased compared to WT (**Figure 4D**). In addition, we found a high degree of sharing for TCRVβ sequences between CD4^+^ TEM and CD8^+^ TCM in KO chimeras. To assess the relative similarity of TCRVβ repertoires in different ways, we further used the VDJTOOLS software to visualize repertoire overlaps of ‘F2’, ‘R’, and ‘D’ metrics. Metric F2 reflects the relative share occupied by the common clonotypes in two groups; Metric R is the overall similarity of repertoire organization; Metric D ignores clonotype frequencies and reflects the number of shared clonotypes between the two groups (25). We found that R and D metrics of CD4^+^ TEM cells and CD8^+^ TCM cells in KO were more diffuse, suggesting higher similarity of shared sequences. F2, R, and D metrics of KO-CD8^+^ TCM samples were more aggregated than WT, indicating different shared clonotypes between the two groups (**Figure 4E**).

### Differences in the Amino Acid Composition of TCRVβ Repertoires Caused by WASp Deficiency

**Figure 5:**
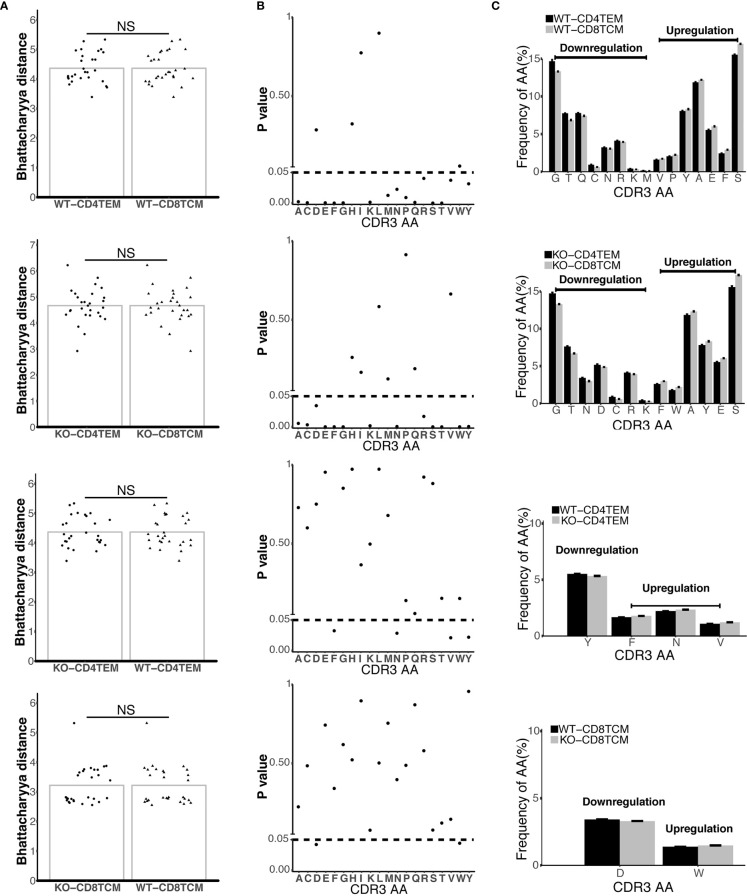
Differential composition of CDR3 amino acids (aa) in TCRVβ repertoires from four compare groups. Bhattacharyya distance analysis for the similarity of CDR3aa from four compare groups: CD4^+^ TEM vs. CD8^+^ TCM in WT chimeras, CD4^+^ TEM vs. CD8^+^ TCM in KO chimeras, WT vs. KO chimeras in CD4^+^ TEM, and WT vs. KO chimeras in CD8^+^ TCM (from up to down) **(A)**. The frequencies of 20 aa in CDR3 from four compare groups **(B)**. Downregulation and upregulation of CDR3 AA in the different groups were shown **(C)**. p < 0.05. NS, No Significant.

To assess the global amino acid composition of TCRVβ repertoires, we used a biological parameter, Bhattacharyya distance, to analyze the similarity between samples at the amino acid level. We found that there was no difference in all of the four compare groups: WT-CD4^+^TEM vs WT-CD8^+^TCM, KO-CD4^+^TEM vs KO-CD8^+^TCM, KO-CD4^+^TEM vs WT-CD4^+^TEM, and KO-CD8^+^TCM vs WT-CD8^+^TCM (**Figure 5A**). Then, we analyzed the usage of each amino acid and found parts of amino acid usage were significantly different among those four groups (**Figure 5B**). When CD8^+^TCM cells were compared with CD4^+^TEM cells, downregulation of Glutamine (Q), Methionine (M) usage and upregulation of Valine (V), Proline (P) usage was specifically found in WT, while downregulation of Aspartic acid (D) and upregulation of Tryptophan (W) usage was specifically found in KO chimeras. In CD4^+^ TEM cells, the usage of Tyrosine (Y) was decreased, and the usage of Phenylalanine (F), Asparagine (N) and V was increased in KO compared to WT. D usage was lower and W was higher when KO-CD8^+^TCM cells were compared with WT (**Figure 5C** **)**.

### WASp Deficiency Did Not Affect the Hydrophobicity and the Length of TCRVβ Sequences

**Figure 6:**
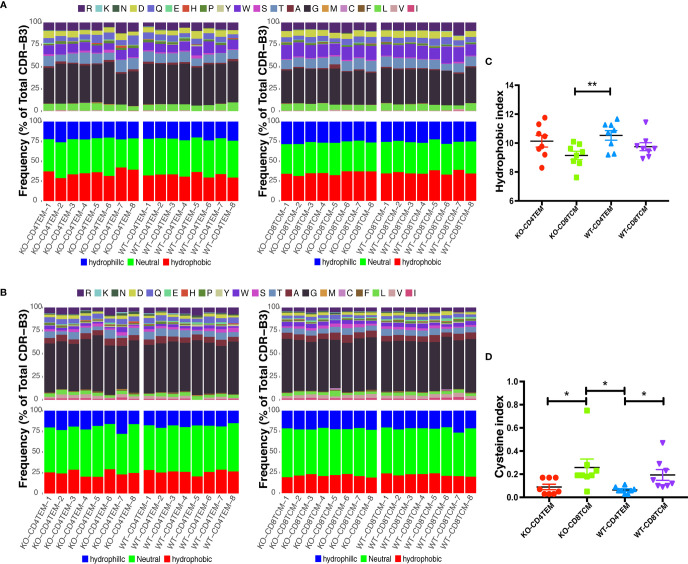
Hydrophobicity of amino acids (aa) in CDR3β repertoires of CD4^+^ TEM cells or CD8^+^ TCM cells from WT and KO chimeras. Composition of aa residues at positions 6 **(A)** and 7 **(B)** of the 13 aa-long CDR3β. Hydrophobic index **(C)** and cysteine index **(D)** in CDR3β repertoires. *p < 0.05; **p < 0.01.

**Figure 7:**
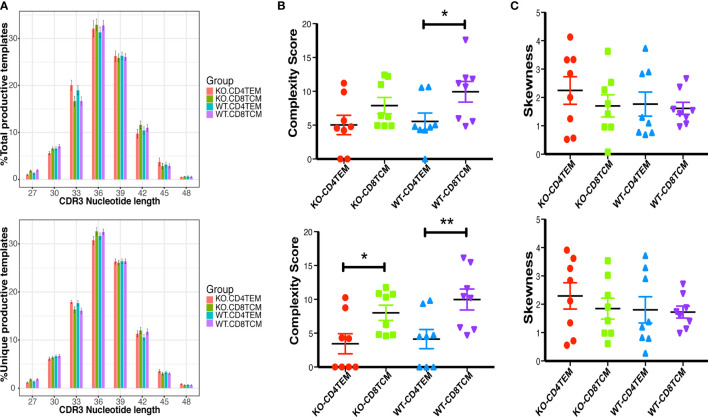
Distribution of the length of CDR3β nucleotide of CD4^+^ TEM cells or CD8^+^ TCM cells from WT and KO chimeras. Distribution of the length of the CDR3β region of TRB total sequences (up) and unique sequences (down) **(A)**. Complexity scores **(B)** and skewness **(C)** of CDR3β total sequences (up) and unique sequences (down). *p < 0.05, **p < 0.01.

Differences in the compositions of amino acids may change the hydrophilicity and hydrophobicity of TCR. The hydrophobicity of TCR and the length of TCRVβ sequences are related to autoimmune diseases, as previously reported (22, 27). About 24 to 72% of WAS patients have autoimmune diseases (28). Therefore, we analyzed the hydrophobicity of amino acids at positions 6 and 7 as reported. And no significant difference was found between KO and WT groups in CD4^+^ TEM cells and CD8^+^ TCM cells (**Figures 6A, B** **)**. A previous study (27) has shown that cysteine and hydrophobic residues in CDR3 serve as distinct T-cell self-reactivity indices. We further calculated the hydrophobic index and cysteine index, and found no significant difference in KO-CD4^+^TEM vs WT-CD4^+^TEM, and KO-CD8^+^TCM vs WT-CD8^+^TCM (**Figures 6C, D** **)**. The results suggest that WASp deficiency did not obviously alter stochastic process of TCR assembly to produce more cysteine and hydrophobic residues in CD4^+^ TEM cells and CD8^+^ TCM cells in relatively young mice. Furthermore, the length of CDR3β nucleotide among the total sequences and unique sequences showed a negative difference again (**Figure 7A**). We then calculated the complexity score and skewness index of total and unique sequences, and they still showed no changes in CD4^+^ TEM cells and CD8^+^ TCM cells between KO and WT (**Figures 7B, C** **)**.

## Discussion

TCR diversity is an essential guarantee for effective T cell immunity. Previous studies have confirmed that TCR diversity can be affected by many factors such as age, pathogen infection, tumor, autoimmune diseases, immunization, and immuno-suppression (18, 19). Limitations to diversity may be a feature of V(D)J rearrangement that is as significant to immune function as the bewildering number of lymphocyte specificities that can theoretically be generated. As proved by other researchers and our team, TCR diversity was severely impaired, mainly in the memory T cell populations in WAS patients (16, 17). However, whether the TCR diversity limitation in WAS was caused by intrinsic WASp deficiency is still unclear. In 2014, Petersen et al. showed that TCR diversity was limited in old WAS^−/−^ mice, but not in young ones. They only detected the total T cells, ignoring that WASp deficiency could selectively affect the diversity of T cell subsets (20). In this study, for the first time, we used WAS chimeric mice model to study the TCR diversity in WAS, which could exclude the potential influence of other WASp deficient immunocytes and other affecting factors. Our work revealed that the limited TCRVβ diversity of CD4^+^ TEM cells and CD8^+^ TCM cells in WAS are intrinsic but not severe. Moreover, WASp-deficiency affected the TCR diversity of CD4^+^ TEM cells more than CD8^+^ TCM cells, indicating WASp may play a more critical role in forming TCR diversity of CD4^+^ TEM cells than that of CD8^+^ TCM cells.

The mechanisms for the limitations to TCR diversity of CD4^+^ TEM cells and CD8^+^ TCM cells in WAS are still unknown. WASp is involved in the process of T cell maturation, differentiation, and proliferation (29, 30). WASp-deficiency affects the maturation and differentiation of T cells inside and outside the thymus. Studies have shown that T cell lymphopenia was common in young WAS patients (31). As there is no limited TCR diversity of naïve T cells in young WAS patients (16), and Petersen et al. had shown that TCR diversity in thymus and spleen was not limited in young WASp^−/−^ mice (20), we also found no limited TCR diversity of naïve CD4 and CD8 T cells in nonchimeric WASp^−/−^ mice (data unpublished), so the impaired TCR diversity of memory T cells may not be related to the deficiency of thymus output. Since WASp-deficiency results in impaired T cell survival and abnormal memory formation efficiency, TCR repertoire analysis of different memory T cells in WAS could provide additional clues regarding the biophysical properties of the TCRs of CD4^+^ TEM cells that may potentially affect the process of memory T cell formation. Due to the limited number of cells, the TCR diversity of CD4^+^ TCM cells and CD8^+^ TEM cells was not studied in this study. Whether the limited TCR diversity of CD4^+^ TEM cells was a continuation of CD4^+^ TCM cells is unknown. CD4^+^ TEM are more susceptible to the effects of WASp deficiency, since its constitutive generation across mouse life likely through TCR driven events. In contrast, CD8^+^ TCM in mice are largely generated in earlier life with a less antigen driven pathway. Even if the mechanisms of their development are not fully understood, it appears to be cytokine-dependent (32). This may explain why few perturbations to TCR repertoire diversity are found in CD8^+^ TCM. Additionally, whether the slightly skewed TCR diversity of CD8^+^ TCM cells could lead to impaired TCR diversity of CD8^+^ TEM cells also remain to be further studied. All in all, the specific mechanism of TCR diversity restriction of memory T cells caused by WASp-deficiency has yet to be further defined.

Comparing V- and J-segment usage frequencies may reflect the functional differences in TCR repertoires and the biases in thymic recombination machinery. This study showed that WASp-deficiency affected the usage of V, D, and J segment genes, which was consistent with previous studies in WAS patients (17). However, we did not find specific V, D, and J segment genes, fixed upregulation, nor downregulation of V(D)J usage in both WAS patients and mice models. So, the difference in the usage of V, D, and J genes caused by WASp-deficiency may randomly happen. Furthermore, the difference in the usage of V, D, and J segments caused by WASp-deficiency was gradually decreased with the combination of V(D)J. Whether the different usage of V, D, and J genes in WAS was related to specific pathogens’ susceptibility or autoimmune diseases still needs more research.

Epidemiological studies showed that 24–72% of patients with WAS had autoimmune diseases, namely, autoimmune hemolytic anemia (AIHA), vasculitis, arthritis, nephropathy, inflammatory bowel disease, and immune granulocytic (28). As reported in type 1 diabetes (24), the highly shared TCR repertoires were enriched in clonotypes with fewer insertions. Our results showed higher sharing of TCRVβ sequences between CD4^+^ TEM and CD8^+^ TCM cells in WAS chimeric mice than in WT, suggesting that WAS chimeric mice are more prone to autoimmunity than WT. However, the segments associated with autoimmune diseases, like TRBV2, TRBV6, and TRBV8.2, were not upregulated in WAS chimeric mice. Since we found no direct association of autoimmunity and V(D)J gene levels, we further detected the hydrophobicity of amino acids at positions 6 and 7, and the length of TCRVβ sequences in amino acid levels. The differences in the compositions of amino acids may change the hydrophilicity and hydrophobicity of TCR, and a previous study showed that the interfacial hydrophobicity of amino acids at positions 6 and 7 of the CDR3β segment robustly promotes the development of self-reactive TCRs (33). Also, the length of TCRVβ sequences is related to autoimmune diseases (22, 27). As a result, although we found differences in the composition of amino acid of TCRVβ repertoires between WAS chimeric mice and the WT, no significant difference in amino acid hydrophobicity at positions 6 and 7 was found. A previous study showed that (27), an increased cysteine index was a specific biomarker of defective cortical tolerance mechanisms, the hydrophobic index appeared more sensitive for detection of a self-tolerance defect but was not specific for either cortical or medullary tolerance mechanisms. Thus the cysteine and hydrophobic indices provide complementary information in the diagnosis and classification of T-cell self-tolerance defects. Therefore, we detected both indices in each cell subpopulation, but there were no significant difference between KO and WT in both CD4^+^ TEM and CD8^+^ TCM cells. This suggested that the WASp defect did not affect the self-tolerance of CD4^+^ TEM and CD8^+^ TCM in thymus. O’Connell et al. reported a change in the length of the TCR sequence in WAS patients (17). However, our data showed no significant change in the length of TCRVβ sequences between WAS chimeric mice and the WT. All these data in our study were insufficient to prove a direct association between the alteration of WAS TCR diversity and autoimmunity. This may be due to an early investigation before the onset of autoimmune disease, which is more often seen in old WASp^−/−^ mice.

The quality of comparative repertoire analysis relies on the TCR library preparation, sequencing (TCR-seq) methods and the following software analysis algorithms (25). This study used 5’ RACE-PCR, deep sequencing, and UMI quantification to the maximum extent to remove technical bias. However, these techniques and methods are still expected to be further optimized. In addition, compared to human samples, the mouse model has similar genetic homogeneity and strengthened repertoire convergence. Therefore, even limited available T cell counts often create the possibility of clear and statistically significant results concerning the characteristics and similarity of syngeneic mouse TCR repertoires for the different T cell subsets, different age groups, and in various transgenic mouse models (25). Although we used chimeric mice to exclude those possible interference factors, there may still be differences between mice and humans, and our findings in mice need further validation in younger WAS patients without infections.

Overall, we confirmed that the effect of WASp-deficiency on the TCRVβ diversity of CD4^+^ TEM cells and CD8^+^ TCM cells was not severe but intrinsic. The intrinsically disturbed TCRVβ diversity in WAS chimeric mice provided clues for researchers to explore the mechanism of autoimmunity and infection in WAS patients. These results also help further study the function of WASp and the specific mechanism of WASp affecting TCR diversity.

## Data Availability Statement

The data presented in the study are deposited in the NCBI bioproject repository, accession number PRJNA792306. (accessible at https://www.ncbi.nlm.nih.gov/bioproject/PRJNA792306).

## Ethics Statement

The animal study was reviewed and approved by the Institutional Animal Care and Usage Committee of Children’s Hospital of Chongqing Medical University.

## Author Contributions

WL performed this research, analyzed data, and wrote the paper. YJ, YW, QZ, LY, TZ, LN, and RD provided help in performing the research. YL reviewed and revised the manuscript. XZ and JW designed the study, reviewed and revised the manuscript. All authors contributed to the article and approved the submitted version.

## Funding

This work was supported by the National Natural Science Foundation of China (81601438).

## Conflict of Interest

The authors declare that the research was conducted in the absence of any commercial or financial relationships that could be construed as a potential conflict of interest.

## Publisher’s Note

All claims expressed in this article are solely those of the authors and do not necessarily represent those of their affiliated organizations, or those of the publisher, the editors and the reviewers. Any product that may be evaluated in this article, or claim that may be made by its manufacturer, is not guaranteed or endorsed by the publisher.
