# Supplementary figures and images for: WASp Deficiency Selectively Affects the TCR Diversity of Different Memory T Cell Subsets in WAS Chimeric Mice
Source: Front Immunol. 2022 Jan 18;12:794795. doi: 10.3389/fimmu.2021.794795 (PMC8803657; doi:10.3389/fimmu.2021.794795)

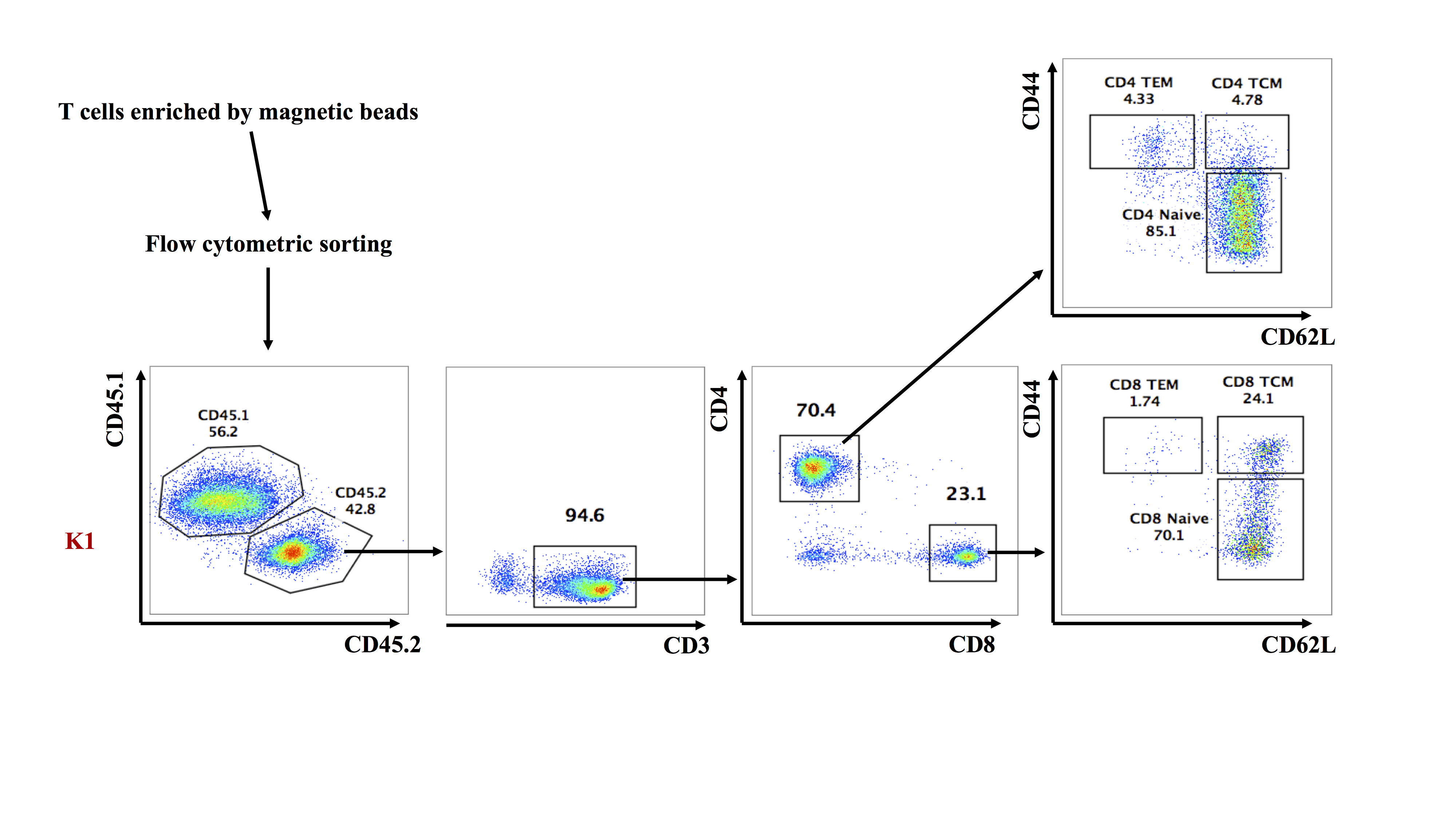

Supplement: Supplementary file 1 [file DataSheet_1.zip › FigureS 1.tiff]

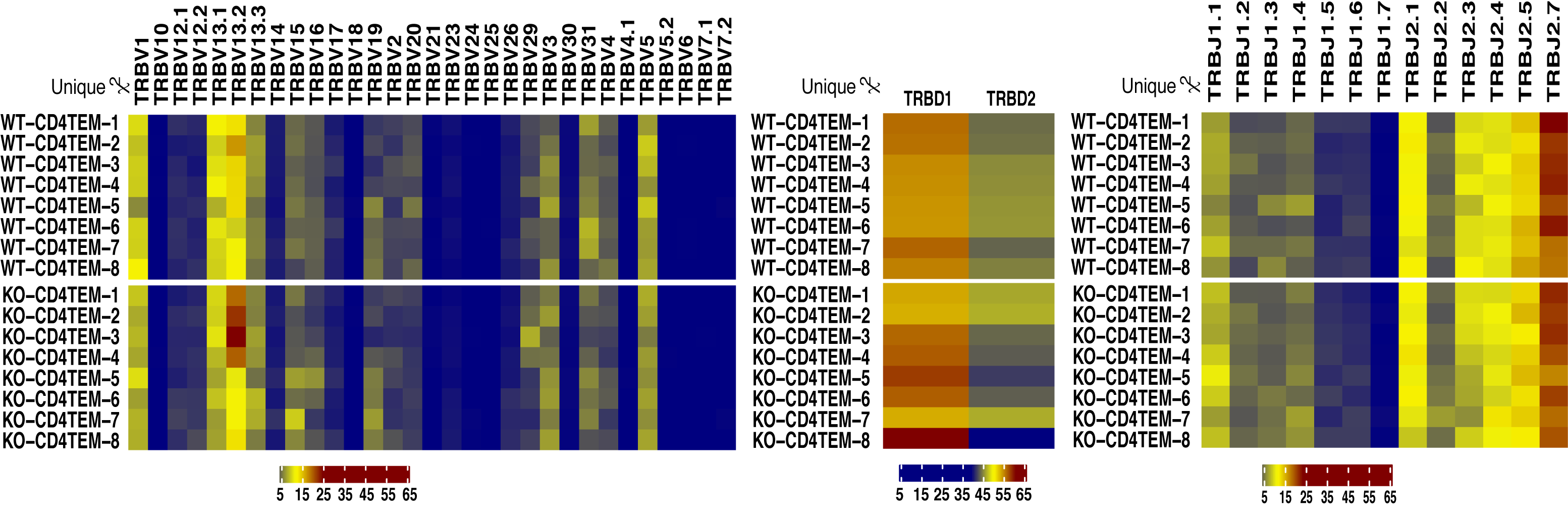

Supplement: Supplementary file 1 [file DataSheet_1.zip › FigureS 2/FigureS 2A.tif]

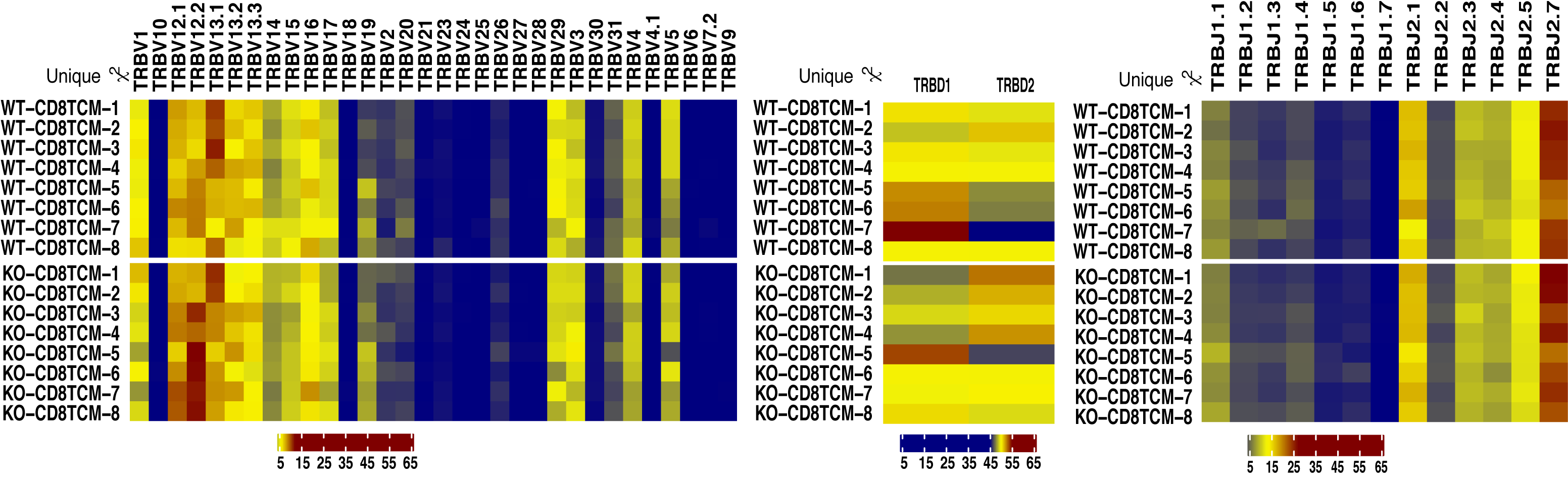

Supplement: Supplementary file 1 [file DataSheet_1.zip › FigureS 2/FigureS 2B.tif]

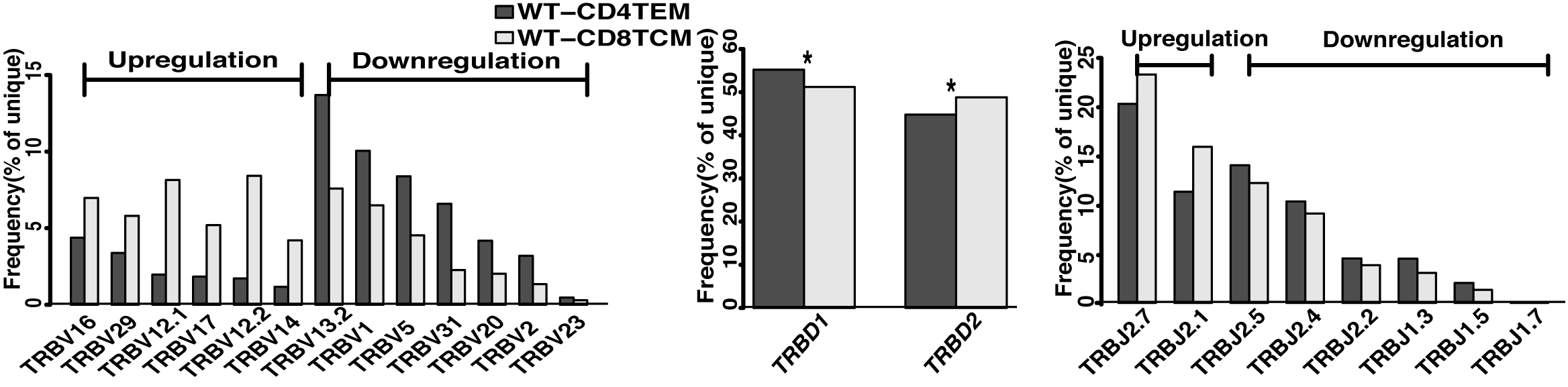

Supplement: Supplementary file 1 [file DataSheet_1.zip › FigureS 2/FigureS 2C.tif]

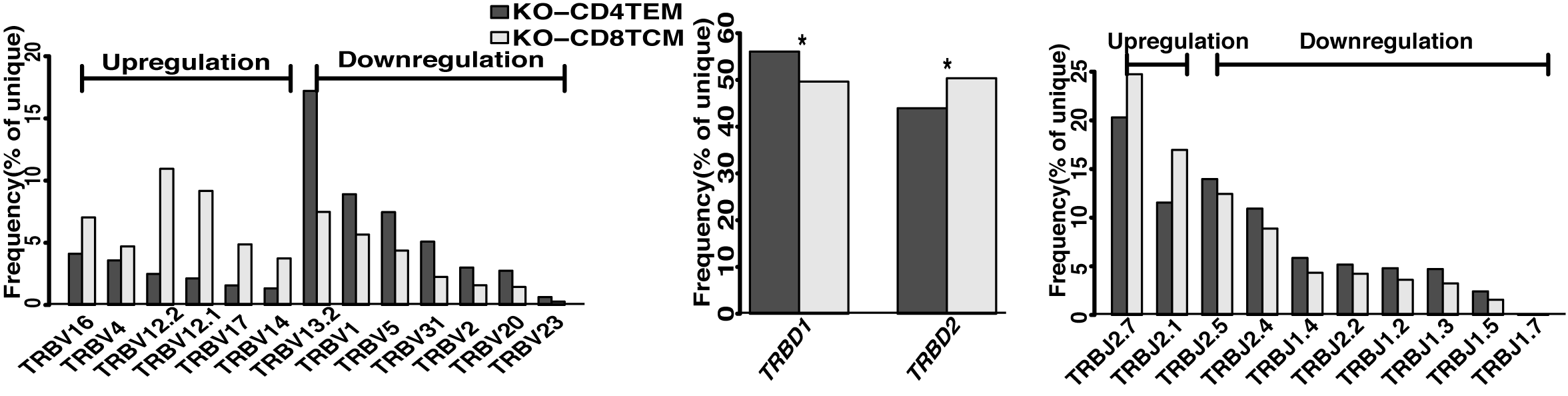

Supplement: Supplementary file 1 [file DataSheet_1.zip › FigureS 2/FigureS 2D.tif]

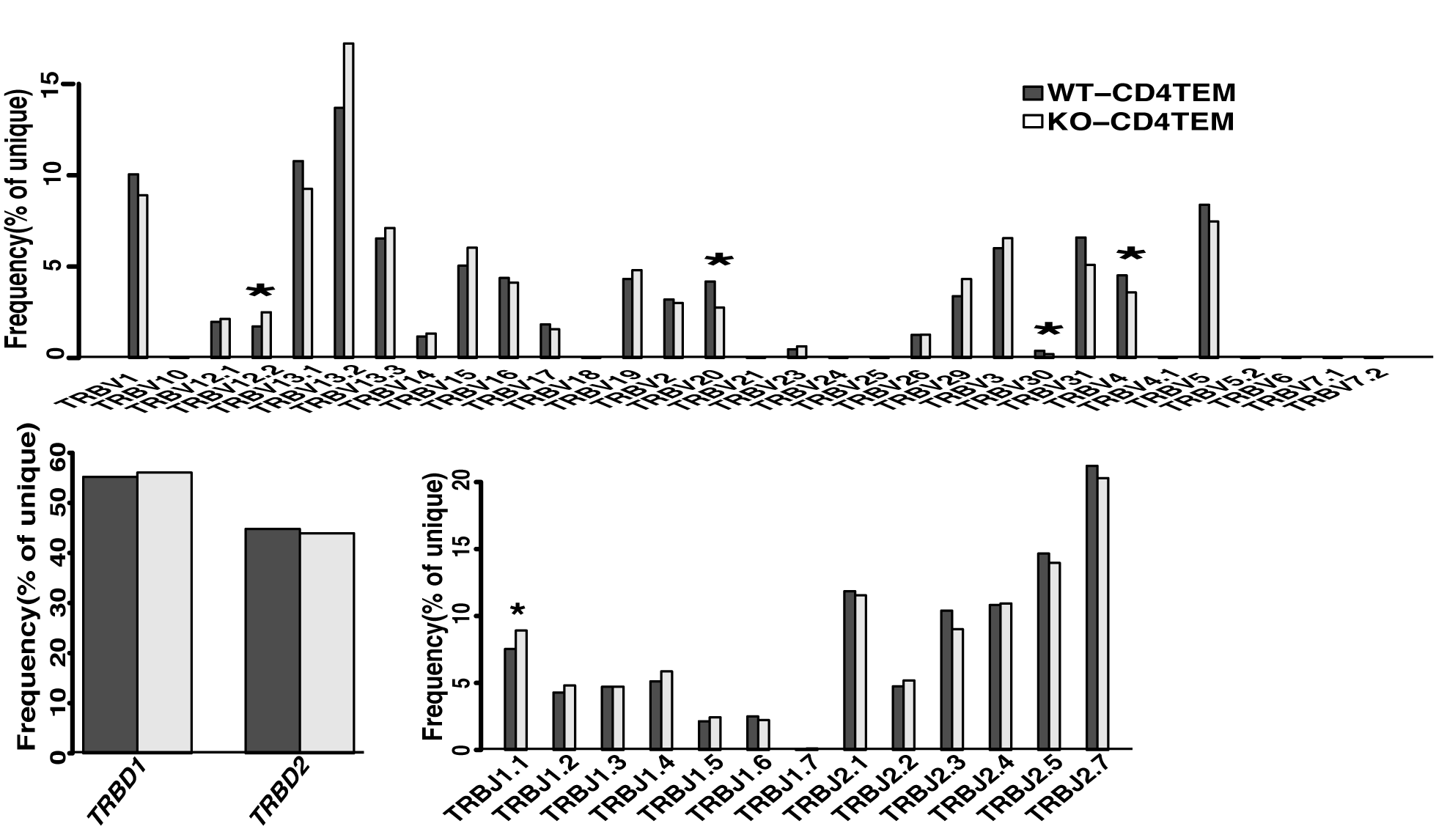

Supplement: Supplementary file 1 [file DataSheet_1.zip › FigureS 2/FigureS2 E.tif]

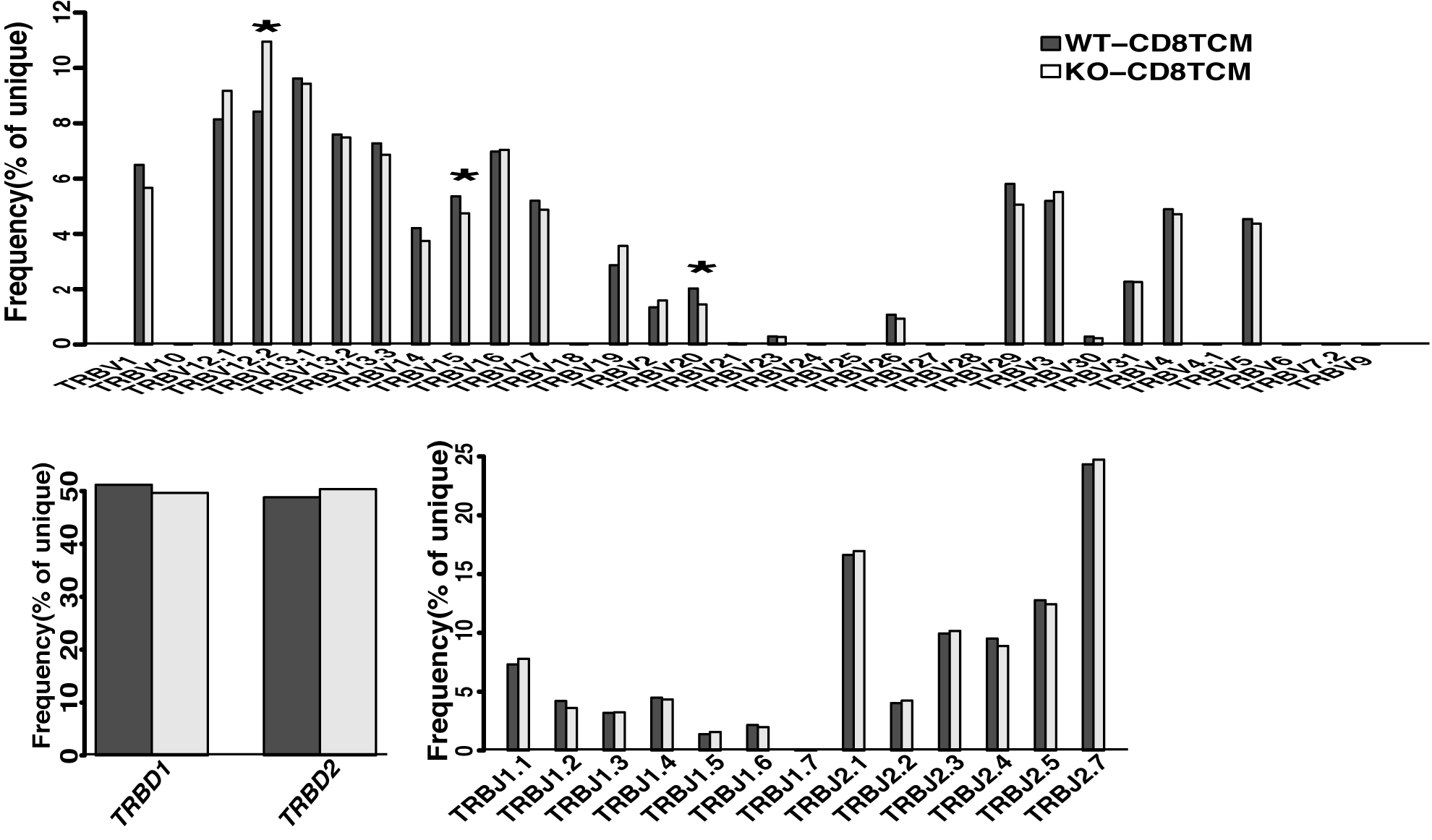

Supplement: Supplementary file 1 [file DataSheet_1.zip › FigureS 2/FigureS2 F.tif]
