# Supplementary material for: WASp Deficiency Selectively Affects the TCR Diversity of Different Memory T Cell Subsets in WAS Chimeric Mice
Source: Front Immunol. 2022 Jan 18;12:794795. doi: 10.3389/fimmu.2021.794795 (PMC8803657; doi:10.3389/fimmu.2021.794795)
Supplement: Supplementary file 2 [file DataSheet_2.docx]

Table S1 Percentage and number of CD4+ TEM and CD8+ TCM in WT and WASKO bone marrow chimeric mice after sorting

| Sample | Percentage of CD4+ TEM in CD3+ T cell | Percentage of CD8+ TCM in CD3+ T cell | The actual number of collected CD4+ TEM  (ten thousand) | The actual number of collected CD8+ TCM  (ten thousand) |
| --- | --- | --- | --- | --- |
| WT-1 | 5.57% | 5.25% | 15.68 | 20.21 |
| WT-2 | 5.88% | 4.15% | 16.18 | 20.63 |
| WT-3 | 6.24% | 4.13% | 16.42 | 20.85 |
| WT-4 | 7.13% | 4.14% | 16.32 | 20.21 |
| WT-5 | 7.21% | 5.68% | 20.7 | 18.83 |
| WT-6 | 7.73% | 6.18% | 20.18 | 12.48 |
| WT-7 | 8.71% | 6.36% | 22.65 | 22.22 |
| WT-8 | 6.41% | 7.21% | 23.31 | 17.45 |
| KO-1 | 3.05% | 5.57% | 14.08 | 18.8 |
| KO-2 | 1.74% | 5.57% | 7.59 | 20.75 |
| KO-3 | 2.17% | 3.07% | 10.51 | 17.41 |
| KO-4 | 2.32% | 6.01% | 10.5 | 20.62 |
| KO-5 | 3.42% | 4.67% | 11.76 | 5.87 |
| KO-6 | 3.20% | 6.37% | 12.93 | 10.28 |
| KO-7 | 2.81% | 7.13% | 10.1 | 8.46 |
| KO-8 | 3.77% | 7.84% | 12.29 | 14.4 |

Table S2 Sequence reads and CDR3 for CD4+TEM and CD8+TCM in WT and WASKO bone marrow chimeric mice

| Sample | Total Reads (Pair) | FilteredReads (total sequences) | ReadUtilization (%) | Clones | Clonetypes | UniqueCDR3 amino acids | UniqueCDR3 sequences |
| --- | --- | --- | --- | --- | --- | --- | --- |
| WT-CD4TEM-1 | 6196002 | 4748550 | 76.64 | 5662 | 3289 | 3289 | 3285 |
| WT-CD4TEM-2 | 6256246 | 4724843 | 75.52 | 6809 | 3555 | 3555 | 3549 |
| WT-CD4TEM-3 | 7383039 | 5484354 | 74.28 | 6003 | 2876 | 2876 | 2866 |
| WT-CD4TEM-4 | 5694544 | 3950786 | 69.38 | 6418 | 3194 | 3194 | 3183 |
| WT-CD4TEM-5 | 4045582 | 3036021 | 75.05 | 2624 | 649 | 649 | 645 |
| WT-CD4TEM-6 | 4392103 | 3854681 | 87.76 | 3502 | 2229 | 2229 | 2222 |
| WT-CD4TEM-7 | 3166244 | 2750879 | 86.88 | 5362 | 2878 | 2878 | 2864 |
| WT-CD4TEM-8 | 3954046 | 2913338 | 73.68 | 2200 | 891 | 891 | 887 |
| WT-CD8TCM-1 | 6701198 | 5105281 | 76.18 | 8463 | 6771 | 6771 | 6766 |
| WT-CD8TCM-2 | 5339753 | 4021570 | 75.31 | 9150 | 7305 | 7305 | 7304 |
| WT-CD8TCM-3 | 6562106 | 4734210 | 72.14 | 9129 | 7012 | 7012 | 7010 |
| WT-CD8TCM-4 | 6457624 | 4656684 | 72.11 | 6625 | 5162 | 5162 | 5162 |
| WT-CD8TCM-5 | 4320384 | 3792408 | 87.78 | 7195 | 5593 | 5593 | 5589 |
| WT-CD8TCM-6 | 3053679 | 2340255 | 76.64 | 2210 | 1075 | 1075 | 1075 |
| WT-CD8TCM-7 | 3466636 | 2739635 | 79.03 | 2050 | 687 | 687 | 686 |
| WT-CD8TCM-8 | 4096326 | 3633584 | 88.7 | 5878 | 5172 | 5172 | 5168 |
| KO-CD4TEM-1 | 6160512 | 4582165 | 74.38 | 7175 | 3022 | 3022 | 3019 |
| KO-CD4TEM-2 | 7997983 | 5829657 | 72.89 | 5669 | 1931 | 1931 | 1924 |
| KO-CD4TEM-3 | 8133354 | 6422271 | 78.96 | 6768 | 2471 | 2471 | 2469 |
| KO-CD4TEM-4 | 7210205 | 5594616 | 77.59 | 5694 | 2071 | 2071 | 2062 |
| KO-CD4TEM-5 | 3991945 | 3474606 | 87.04 | 3756 | 1201 | 1201 | 1192 |
| KO-CD4TEM-6 | 3091268 | 2446609 | 79.15 | 2751 | 1245 | 1245 | 1244 |
| KO-CD4TEM-7 | 6429432 | 5595099 | 87.02 | 1438 | 616 | 616 | 610 |
| KO-CD4TEM-8 | 4594889 | 3913616 | 85.17 | 3348 | 1492 | 1492 | 1486 |
| KO-CD8TCM-1 | 8538488 | 6872499 | 80.49 | 8143 | 6870 | 6870 | 6868 |
| KO-CD8TCM-2 | 7416822 | 5793611 | 78.11 | 8120 | 6754 | 6754 | 6754 |
| KO-CD8TCM-3 | 7243919 | 5640005 | 77.86 | 6178 | 4295 | 4295 | 4295 |
| KO-CD8TCM-4 | 9000316 | 7197342 | 79.97 | 7587 | 5941 | 5941 | 5937 |
| KO-CD8TCM-5 | 4424167 | 3305488 | 74.71 | 2236 | 801 | 801 | 799 |
| KO-CD8TCM-6 | 3805857 | 3012573 | 79.16 | 3047 | 1623 | 1623 | 1622 |
| KO-CD8TCM-7 | 3640802 | 3211601 | 88.21 | 4357 | 3337 | 3337 | 3332 |
| KO-CD8TCM-8 | 3526218 | 2997821 | 85.02 | 4467 | 3446 | 3446 | 3444 |

**Figure Legends**

Figure.S1. Isolation of T cell subsets in bone marrow chimeric mice by magnetic beads and flow cytometric sorting.

Figure.S2. Differential usage of V, D and J genes in the unique sequences of *TRB* repertoires of CD4^+^ TEM and CD8^+^ TCM in WT and KO chimeric mice. Heatmap represents V, D and J gene usage frequency in unique *TRB* sequences of CD4^+^ TEM (a) and CD8^+^ TCM (b) in WT and KO chimeras. Relative frequency of usage of TRBV, TRBD, TRBJ gene segments of CD4^+^ TEM vs. CD8^+^ TCM in WT chimeras (c), of CD4^+^ TEM vs. CD8^+^ TCM in KO chimeras (d), of WT vs. KO chimeras in CD4^+^ TEM (e), and of WT vs. KO chimeric mice in CD8^+^ TCM (f). * p < 0.05.
